# Metabolic Reprograming of Microglia in the Regulation of the Innate Inflammatory Response

**DOI:** 10.3389/fimmu.2020.00493

**Published:** 2020-03-20

**Authors:** Clotilde Lauro, Cristina Limatola

**Affiliations:** ^1^Department of Physiology and Pharmacology, Sapienza University of Rome, Rome, Italy; ^2^Laboratory Affiliated to Istituto Pasteur Italia– Fondazione Cenci Bolognetti, Department of Physiology and Pharmacology, Sapienza University of Rome, Rome, Italy; ^3^IRCCS NeuroMed, Pozzilli, Italy

**Keywords:** microglia, metabolism, neuroinflammation, neurodegeneration, homeostasis

## Abstract

Microglia sustain normal brain functions continuously monitoring cerebral parenchyma to detect neuronal activities and alteration of homeostatic processes. The metabolic pathways involved in microglia activity adapt at and contribute to cell phenotypes. While the mitochondrial oxidative phosphorylation is highly efficient in ATP production, glycolysis enables microglia with a faster rate of ATP production, with the generation of intermediates for cell growth and cytokine production. In macrophages, pro-inflammatory stimuli induce a metabolic switch from oxidative phosphorylation to glycolysis, a phenomenon similar to the Warburg effect well characterized in tumor cells. Modification of metabolic functions allows macrophages to properly respond to a changing environment and many evidence suggest that, similarly to macrophages, microglial cells are capable of a plastic use of energy substrates. Neuroinflammation is a common condition in many neurodegenerative diseases and the metabolic reprograming of microglia has been reported in neurodegeneration. Here we review the existing data on microglia metabolism and the connections with neuroinflammatory diseases, highlighting how metabolic changes contribute to module the homeostatic functions of microglia.

## Introduction

### Microglia Phenotypes and Metabolic States

Microglia are the resident immune cells of the central nervous system (CNS) and, depending on the brain region, they can represent from 5 to 12% of total cell population (1). Microglial cells continuously monitor the surrounding parenchyma to sense alteration of brain functions (2, 3) and are involved in controlling neuronal excitability, synaptic activity, neurogenesis, and clearance of apoptotic cells in the healthy adult brain (4). Microglia interact with the cerebral microenvironment through different molecules such as chemokines, cytokines, and trophic factors which, in turn, modulate microglia activities converting the homeostatic microglia into reactive microglia and *viceversa* (5). Alterations of functional phenotype are accompanied by dynamic changes of shape of cell body and processes, although no unique correlation among microglial cell morphology and functional phenotype has been identified (6). However, in early stages of brain development, and upon *in vitro* activation with pro-inflammatory stimuli, such as bacterial lipopolysaccharide (LPS), microglial cells display an ameboid profile, with large and round cell bodies, short and thick branches; this morphology is often accompanied by an increased phagocytic activity, production of specific molecules and gene expression signatures. At more mature stages of development, microglia have usually a highly ramified morphology, dynamically reacting to brain parenchymal alterations and injuries (3) and changing phenotype from *surveillant* to pro- or anti-inflammatory in response to pathological conditions (7, 8). Under pathological conditions, it was shown that microglia comprise cells with diverse phenotypes (9). In fact, microglial-activated cells can be roughly divided into classically activated M1 cells, with cytotoxic and pro-inflammatory properties and alternatively activated M2 cells, with phagocytic activities. The M2 condition can be further divided into three classes: M2a, involved in repair and regeneration; M2b, an immune-regulatory phenotype; M2c, an acquired-deactivating phenotype (10, 11). Indeed, more recent transcriptomic analysis of microglia in different brain area and different disease conditions, reveal a much higher complexity, with several overlapping genes and few signature genes specifically expressed by microglia subgroups (12, 13). Upon aging, microglia phenotype changes further, and it was recently demonstrated an age-related senescent microglial phenotype in humans, possibly involved in pathological processes associated with brain aging (14). Like other cells, in order to perform their functions, microglia require a large amount of energy and it has been recently shown that different microglia phenotypes are associated with distinct metabolic pathways (15–18). Under normal oxygen supply, cells produce energy in the mitochondria, in the glycolytic pathway, through the oxidative phosphorylation (19); in hypoxic conditions, the anaerobic glycolysis converts pyruvate into lactate in the cytoplasm (20, 21). The bioinformatics analysis of a transcriptome database of mouse brain cells (22) showed that microglia express all the genes required for the glycolytic and the oxidative energy metabolism (16). It has been proposed that glucose metabolism exerts transcriptional control over microglial activation, and that the homeostatic phenotype of (cultured) microglia preferentially utilize oxidative metabolism (23–26). An essential fuel for microglia is glucose, which enters the cell through different transporters (GLUTs) (27). Microglia predominantly express GLUT3 (28) and the fructose transporter GLUT5 (29, 30), but under inflammatory conditions, GLUT1 expression is upregulated to increase glucose uptake and promote glycolysis (31). In the absence of glucose, microglia are able to use free fatty acids as alternative energy source, as also suggested by the accumulation of lipid droplets in glucose-deprived microglial cells (32). Microglia also express the nicotinamide adenine dinucleotide phosphate (NADPH) oxidase NOX2 and the superoxide is used to kill pathogens (33, 34). Glucose metabolism controls NOX activation by the NADH-dependent transcriptional co-repressor C-terminal binding protein (CtBP) that affects nuclear factor kappa-light-chain-enhancer of activated B cell (NF-κB) signaling and the expression of inducible nitric oxide synthase (iNOS) (35, 36). Interestingly, microglia also express the monocarboxylic transporter (MCT) 1 and 2 and absorb lactate and ketons (37) and it has been demonstrated that a ketogenic diet is correlated with a suppression of microglia activation (38–40) likely due to the inhibition of histone deacetylases (HDACs) by ketonic bodies, which decreases NK-kB signaling (41–43). Moreover, silencing HDAC activity affects microglia during development and in adulthood, as a function of the activation state, suggesting that epigenetic changes affect cellular metabolism in activated microglia, modulating microglia function (44). Microglial activity, together with glucose availability and glycolytic rate, influences pro-inflammatory gene and protein expression (45). The oxidative phosphorylation occurs within the mitochondria and produces more ATP molecules; on the other hand, glycolysis permits a faster ATP production in activated microglia (46) allowing a rapid metabolism for cell growth, and the production of cytokines and reactive oxygen species (47). These pathways of energy production are both of primary importance for microglia to maintain their homeostatic functions and are critical for the progression and repair mechanisms upon CNS injury and neurodegeneration.

### The “Warburg Effect” in Microglia

It is well-established that peripheral immune cells, such as macrophages and dendritic cells (DCs), switch from the oxidative phosphorylation to the aerobic glycolytic pathway when activated (48–50), similarly to what described in tumor cells (Warburg effect) (51–53), to foster cell proliferation. Even if microglia originate from a distinct embryological lineage, they share many characteristics with macrophages (54), as concern cell plasticity and the adaptable use of energy substrates. Several reports recently marked the metabolic similarity of microglia with DCs and macrophages: microglia exposed to inflammatory stimuli exhibit a transient upregulation of specific metabolic pathway's genes (45), indicating that energy metabolism is modulated during brain inflammation. Many studies have been performed with microglia cell lines: in particular, it was observed that upon activation, microglia alter the mitochondrial metabolism in a nitric oxide (NO)-dependent manner (24, 25). Another study demonstrated that lysophosphatidic acid (LPA) stimulates alteration in glycolysis, morphology and motility of C13NJ microglia cells (23). Furthermore, lipopolysaccharide (LPS) stimulation of the murine microglial cell line BV-2 increased lactate production, reduced the mitochondrial oxygen consumption and ATP production, with the resulting increase of glycolysis and decrease of oxidative phosphorylation (15), ultimately increasing nucleic acid production for gene transcription (55). It has also been observed that treatment of primary microglia with Deoxy-D-glucose (2-DG), a blocker of glycolytic pathway, reduced tumor necrosis factor α (TNFα) and interleukin-6 (IL-6) production through NF-kB inhibition, leading to microglia death (56, 57). On the other hand, primary rat microglia cultured with increasing glucose concentration (from 10 to 50 mM) boosted TNFα secretion (58, 59). More recently, Rubio-Araiz et al. showed that primary microglia exposure to LPS and amyloid-β (Aβ) induced an inflammatory state associated with the increase of the glycolytic enzyme 6-phosphofructo-2-kinase/fructose-2,6-biphosphatase 3 (PFKFB3), with a boost in extracellular acidification rate (ECAR) (60). IFNγ and Aβ also increased microglia glycolysis together with an increase in PFKFB3, hexokinase II and Pyruvate kinase isozymes M2 (PKM2) (61), suggesting that inflammation affects microglia metabolism, driving the glycolysis pathway through increased PFKFB3 activation. Consistently, classic anti-inflammatory stimuli, such as interleukin-4 (IL-4), decreased glucose consumption and lactate production (55) in BV2, and this was confirmed in primary microglia, where IL-4 increased oxygen consumption rate (OCR), basal respiration and ATP production (62); in addition, IL-4/IL-13 stimulation maintained an oxidative metabolic state (16), suggesting that this metabolic shift was associated with a reduced need for anabolic reactions. Pro-inflammatory activation of microglia leads to changes in mitochondrial dynamics and in particular to the metabolic switch from oxidative phosphorylation to glycolysis. It has been recently demonstrated that in inflammatory conditions, microglia upregulate GLUT1 to facilitate glucose uptake and promote glycolysis and that the blockade of GLUT1 reprogrammed back microglia from glycolysis to mitochondrial oxidative phosphorylation, thus altering microglial activation and reducing retinal neurodegeneration in a mouse model (31). These changes represent an adaptive mechanism, since the conversion of microglia from surveying to reactive is accompanied by increased energy consumption. In line with this view, Nair et al. showed that LPS-treated primary microglia increased mitochondrial fragmentation together with a reduction in oxidative phosphorylation and an increase in both oxygen consumption rate, glycolysis and cytokine production (63). In fact, fragmented mitochondria represent the preferred morphofunctional state when the respiratory activity is low (64). Moreover, when mitochondrial fragmentation increases, due to overmuch fission, it can increase the inflammatory response of microglia modulating DRP1 de-phosphorylation and ROS elimination, as already demonstrated for macrophages (65, 66). The same authors also demonstrated that normalizing mitochondrial membrane potential and ROS production with a putative mitochondrial division inhibitor (Mdivi-1) abolished the release of pro- and anti- inflammatory cytokines and chemokines (63). It fact, it has been shown that LPS induces an increase in proton leak and in membrane potential of primary microglia, partially mediated by the uncoupling proteins (UCPs) present in the mitochondrial inner membrane (67).

### Microglia Dysfunction and Neurodegenerative Diseases

When exerting homeostatic activities, microglia rely on several membrane proteins: the Pattern Recognition Receptors (PRRs) and immune receptors such as the triggering receptor expressed on myeloid cells-2 (TREM2), the signal regulatory protein 1A (SIRP1A), the fractalkine receptor (CX3CR1), the cell surface transmembrane glycoprotein receptor CD200 (CD200R) and the colony stimulating factor 1 receptor (CSF-1R) (68–70) that recognize Damage-associated molecular patterns (DAMPs) or Neurodegeneration-associated molecular patterns (NAMPs) (71). Upon stimulation by potentially dangerous molecules, microglia assume a neurodegenerative phenotype (MGnD) or disease-associated microglia (DAM), also recently identified as “dark microglia” (72) in several neurodegenerative diseases such as amyotrophic lateral sclerosis (ALS), multiple sclerosis (MS), and Alzheimer's disease (AD) (71, 73, 74). It was shown that an aberrant microglia activation may result in a loss or alteration of their physiological functions with possible implications on the emergence or maintenance of pathological conditions; moreover, neuro-inflammation caused by microglia hyperactivity has been associated with several neurodegenerative diseases (12, 75–77) and many evidence support a metabolic reprograming of microglia in neurodegeneration (17). A possible mechanism explaining this microglial metabolic reprogramming has been described in a mouse model of AD, where Aβ directly triggers microglial inflammation together with a metabolic reprogramming from oxidative phosphorylation to glycolysis, in mTOR-HIF-1α pathway-dependent manner (78). Upon activation, microglia enter in a tolerant state with defects in cellular metabolism and reduced responses to inflammatory stimuli, including cytokine secretion and phagocytosis, suggesting that Aβ-induced microglial tolerance might represent a critical cue for AD progression (78). Nonetheless, when microglial glycolytic metabolism was reactivated by interferon-γ (IFN-γ) treatment, which is a known regulator of the mTOR (79) and glycolysis pathway (80), the phagocytic activity of microglia was restored, Aβ plaques and neuronal losses were reduced and cognitive impairment was rescued (78) indicating a (close) relation between the cellular metabolic pathways and functional phenotypes of microglia. The involvement of mTOR pathway in modulating microglial metabolism in AD was also previously suggested by Ulland et al., that identified TREM2 and the downstream mTOR signaling as mediators in maintaining microglial metabolic homeostasis (17). In particular they found that in AD patients carrying a TREM risk variant (81, 82) and in TREM2-deficient mice with AD-like pathology, microglia have an anomalous autophagy activity due to defective mTOR signaling. They demonstrated that upon AD development, TREM2 deficiency affects the mTOR pathway and the energetic metabolism in microglia: TREM2 deficiency was associated with decreased expression of genes for glucose transporters, glycolytic enzymes, and the transcription factor HIF1α, all involved in glycolysis (17). The role of TREM2 in microglial metabolic function was also confirmed in microglia produced by patient-derived iPSC expressing loss of function variants of TREM2: TREM2 variants could not perform the immune-metabolic switch toward glycolysis due to altered PPARg-p38MAPK-PFKFB3 signaling (83). Of note, in AD as well as in other diseases such as traumatic brain injury and ischemia, microglia phenotype changes from anti- to pro-inflammatory upon disease progression (84–86). In particular, in brain ischemia, a phenotypic change is well-documented (87, 88): few minutes after the ischemic attack, resident microglial cells, mainly in the peri-infarct region, acquire an anti-inflammatory phenotype in order to restrain brain damage. Few days after the ischemic insult, pro-inflammatory microglia predominate in the region adjacent the infarct zone (89, 90) and release ROS and pro-inflammatory cytokines that induce the activation of cerebrovascular endothelial cells and sustain the adhesion and transmigration of leukocytes into the injured tissue, contributing to further brain damage (91–94). It was recently demonstrated that upon permanent middle cerebral artery occlusion (pMCAO), the expression profiles of anti- and pro-inflammatory genes in microglia correlates with the expression of genes related to the oxidative and glycolytic pathway, respectively (18), suggesting that a targeted modulation of microglia could be used to reduce the extent of tissue damage in brain ischemia. All these data indicate that a metabolic reprogramming is crucial for microglial function in several neuropathologies and the identification of tools to modulate microglial bioenergetics pathways might be a promising therapeutic strategy.

### Microglia Metabolic Remodeling as Therapeutic Approach

**Figure 1:**
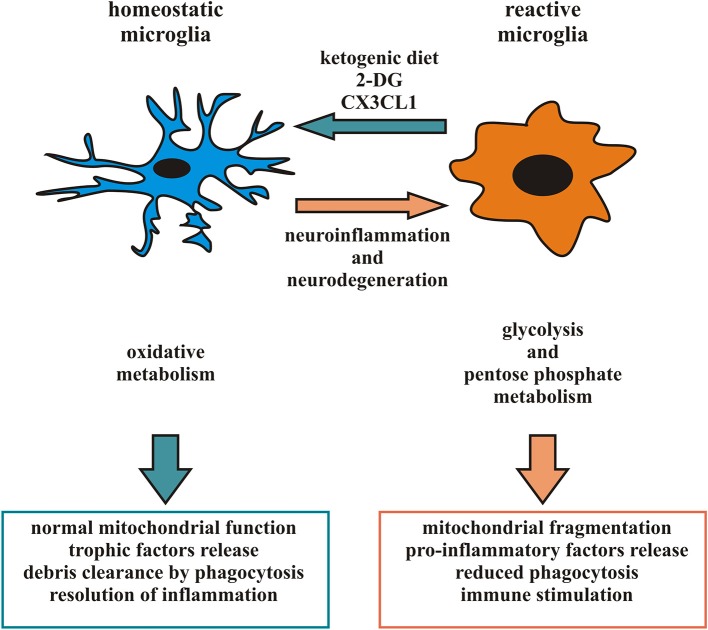
Microglia phenotype and metabolic state: in response to appropriate signals, reactive microglia can switch from a pro-inflammatory to an anti-inflammatory phenotype and vice versa, reorganizing their structure and functions. In particular, pro-inflammatory microglia release cytokines and free radicals that impair brain repair and regeneration while anti-inflammatory microglia resolve cerebral inflammation and promote brain repair increasing phagocytosis and release of trophic factors. Different phenotypes of microglia are associated to distinct metabolic pathways, in order to perform their different functions and their activation leads to changes in mitochondrial dynamics and switch among oxidative phosphorylation and glycolytic metabolism. Several neurodegenerative diseases have been associated with neuro-inflammation related to microglia hyperactivity or mitochondrial dysfunction. Factors able to promote an anti-inflammatory microglia, such as a ketogenic diet, 2-DG and CX3CL1, may represent an intriguing approach to counteract some aspect of neurodegenerative diseases.

Considering the heterogeneity of microglia phenotypes present in specific time windows in different CNS regions in pathophysiological conditions (87, 88, 95), studies based on general microglial depletion cannot be considered effective therapeutic strategies to eliminate potentially dangerous microglia phenotypes. Accordingly, since a given microglia subpopulation can plastically modify its phenotype and function in response to signals from the microenvironment (10), the targeting of specific microglial phenotypes in a proper time window could represent a more selective and efficacious approach and represent the current challenge of this field of research. One recent experimental approach proposes to induce a ketogenic state in microglia, suppressing glucose utilization to reduce inflammation, tissue loss and functional impairment after brain injury (41–43). The activation of the G-protein-coupled receptor 109A (GPR109A) with b-hydroxybutyrate (41, 43, 96) on microglial cells attenuates the NF-kB signaling and the production of pro-inflammatory cytokines, promoting a microglial neuroprotective phenotype in a mouse model of PD (42). Also, a metabolic switch toward oxidative metabolism might contribute to promote a protective microglia in some pathophysiological conditions, resulting in the production of metabolites beneficial for neurons (18, 97). Starting from the observation that in animal models of cerebral ischemia the increased anti-inflammatory polarization of microglia is associated with a smaller infarct area and the resolution of inflammation (98) it could be useful to identify a number of factors able to induce a metabolic switch in favor of an anti-inflammatory state of microglia. Among the possible candidates is CX3CL1, a chemokine released from neurons in response to ischemic insult that has neuroprotective properties in permanent focal cerebral ischemia (99), able to modulate the activation state of microglia and its metabolism, down-modulating the expression of several pro-inflammatory and glycolytic pathway-related genes and inducing an increase in the expression of several anti-inflammatory and oxidative pathway-related genes after the ischemic insult (18). CX3CL1 thus acts potentiating the anti-inflammatory function of microglia, prolonging this phenotype to limit neuro-inflammation and gaining time used by parenchymal cells to organize a neuroprotective response. Another possibility could be to regulate the dynamic of microglial mitochondria to prevent neurological disorders caused by aberrant microglial activation: as discussed above, microglia mitochondrial functions correlate with neuronal survival, as a function of microglial ROS production, but also indirectly affecting the activation state and cytokine production (63, 65). Therefore, targeting cytokines that promote the anti-inflammatory phenotype of microglia may result in protecting mitochondrial homeostasis and, on the other hand, direct approaches to enhance microglial mitochondrial function may promote the activation of the microglia anti-inflammatory state (Figure 1).

## Conclusion

Many brain disorders are accompanied by changes in energy metabolism (100–105). While the mechanisms connecting inflammation to cell energy metabolism have been addressed (106), few information are available on how energy metabolism affects the inflammatory responses. Since microglia represent the sentries of the CNS, consistently, they respond to changes in brain metabolism; however, very little is known about their own metabolism, especially because most of the metabolic studies in microglia were conducted in dissociated populations of primary cultures, which do not mirror the complexity and diversity of multiple cell types which interact with other cells and external cues to adapt to bioenergetics changes. For this reason, it is essential to identify experimental approaches to study microglia metabolism in *in vivo* systems, in pathophysiological conditions. Moreover, most of our knowledge on microglia biology derives from rodents and, even if some *in vitro* studies suggest that polarization of human microglia might resemble that observed in rodents cells (107), there are several important differences between rodent microglia and their human counterparts (108) and additional studies using human biological systems, such as induced pluripotent stem cells, will be useful in the effort to translate the studies on microglia phenotype into preclinical biomedical research. However, despite these limitations, microglia represent an intriguing target for the treatment of neurodegenerative diseases and targeting their metabolism in order to change their immunological phenotype could represent a promising future therapeutic approach.

## Author Contributions

CLa made substantial contributions to conception and design of the review. CLi contributed to the manuscript revision, read, and approved the submitted version.

### Conflict of Interest

The authors declare that the research was conducted in the absence of any commercial or financial relationships that could be construed as a potential conflict of interest.
